# Melatonin and Grain Legume Crops: Opportunities for Abiotic Stress Tolerance Enhancement and Food Sustainability

**DOI:** 10.3390/plants14213324

**Published:** 2025-10-30

**Authors:** Humberto A. Gajardo, Jorge González-Villagra, Patricio Arce-Johnson

**Affiliations:** 1 Instituto de Ciencias Aplicadas, Facultad de Ingeniería, Universidad Autónoma de Chile, Temuco 4780000, Chile; 2 Escuela de Agronomía, Facultad de Ciencias, Ingeniería y Tecnología, Universidad Mayor, Temuco 4801043, Chile; jorge.gonzalez@umayor.cl; 3 Instituto de Ciencias Aplicadas, Facultad de Ingeniería, Centro de Investigación e Innovación, Universidad Autónoma de Chile, Avenida del Valle 534, Huechuraba, Santiago 8581151, Chile

**Keywords:** melatonin, legumes, abiotic stress, biostimulants

## Abstract

Grain legume crops are rich in nutritional value and play a crucial role in global food sustainability. Like many other crops, they are affected by various abiotic stresses that reduce yield and seed quality, thereby threatening food security. Several strategies have been proposed to mitigate these effects and enhance yield. Among them, the use of biostimulants offers a sustainable and efficient approach to improving stress tolerance in the short term. However, the molecular mechanisms underlying the effects of individual or combined molecules remain poorly understood and could significantly influence the development of edited crops with enhanced stress tolerance in the long term. Melatonin (MT) has emerged as a versatile biostimulant, providing multiple benefits across different crop species. Given its key role in plant physiological processes, along with endogenous production, receptor identification, and signaling functions, it has been suggested to act as a hormone-like molecule. Nonetheless, the molecular response triggered by its application remains under investigation, particularly in grain legume species. This review explores the current state of MT applications for alleviating abiotic stress in grain legume crops, with emphasis on drought, salinity, metals/metalloids, and heat stress. We integrate biochemical, molecular, and physiological evidence to highlight the main scientific gaps regarding MT function in grain legumes. Finally, we discuss the biotechnological prospects of combining MT with modern breeding tools, as well as strategies for its delivery and sustainable production.

## 1. Introduction

Grain legume crops are an important source of nutrients in the human diet, particularly in low-income and developing countries. In 2023, the harvested area of pulses reached 96 million hectares, with a production of 94 million tons [1]. These staple foods, together with cereals, will be essential to meet human food requirements in the coming years, when significant population growth is expected [2]. Legumes are rich in proteins, minerals, essential amino acids, dietary fiber, vitamins, and bioactive compounds, making them highly nutritious foods [3], with the potential to prevent chronic diseases such as obesity, type II diabetes, and cardiovascular disorders [4]. However, as in many other crops, the yield and sustainability of grain legumes are threatened by multiple abiotic stressors, such as drought, salinity, metal and metalloid soil contamination, and heat, among other increasingly recurrent factors. Indeed, these stressors can occur simultaneously or sequentially, producing devastating effects on smallholder and family farming systems [5]. To address this scenario, multiple strategies have been implemented in grain legume crops to select and release varieties more adapted to fluctuating climatic conditions or to protect elite varieties from stressful environments. These strategies include classical and marker-assisted breeding platforms [6], transgenic approaches, varieties developed through genome-editing tools such as CRISPR/Cas [7], and the use of biostimulants to improve agronomic performance and seed quality under stress [8]. Additionally, efficient agronomic management practices, such as irrigation [9] and crop rotation systems [10], are fundamental to improving grain legume crop production worldwide.

Currently, the use of biostimulants in agriculture as a sustainable strategy to mitigate the detrimental effects of abiotic stressors on crop yield is under continuous investigation. The diversification of biostimulants includes plant growth-promoting microorganisms, humic substances, phenolic compounds, amino acids, seaweed polysaccharides, and plant hormones [11,12,13]. Among the most studied elicitor molecules, plant hormones such as absicic acid (ABA), salicylic acid (SA), jasmonates (JA), gibberellins (GA), cytokinins (CK), brassinosteroids (BR), and strigolactones (SL) have shown great potential to increase abiotic stress tolerance by modulating central physiological and cellular processes in different plant species [14].

In recent years, scientific evidence has suggested that melatonin (N-Acetyl-5-methoxytryptamine), a biogenic indole amine, plays a role in maintaining plant cellular homeostasis. Melatonin (MT) is a naturally occurring molecule identified in eukaryotic organisms (animals, plants, algae) and in prokaryotes (bacteria). In plants, MT has been reported to participate in several physiological processes, including germination [15], root growth [16], stomatal regulation [17], fruit ripening [18], and stress responses [19]. In this context, MT has been described as a novel plant hormone with pleiotropic effects. Under abiotic stress, this molecule acts as a hormonal compound and/or regulator of several cellular processes in plants, such as protecting and stimulating photosynthesis [20,21], inducing enzymatic antioxidant responses, and promoting the synthesis of osmoprotectant compounds [22]. The discovery of the MT receptor PMTR1 in guard cells of *Arabidopsis* and *Vicia faba* opened new avenues to understanding its molecular role in stomatal regulation [23]. Furthermore, MT applications in *Malus prunifolia*, *Malus hupehensis*, and *Zea mays* under drought conditions have been shown to downregulate ABA biosynthetic components, thereby inducing stomatal opening and restoring photosynthesis [24,25,26]. Otherwise, contradictory results have been reported in *Arachis hypogaea,* through the assessment of the same ABA-related genes [27], while in fodder soybean (*Glycine max* L.), MT triggered ABA-independent drought resistance mechanisms [28]. Moreover, interactions with other cellular components or cis-regulatory elements that explain its pleiotropic effects on physiological processes remain elusive. Moreover, despite growing evidence on the benefits of MT applications in alleviating abiotic stress in different species [20,29], the regulation of endogenous MT production, its interaction with other hormones, and its molecular mechanisms of action still need to be elucidated. This review aims to summarize the current knowledge on the use of MT in grain legume crops to alleviate abiotic stress, highlight the main insights into its molecular mechanisms, and discuss biotechnological prospects for future research on this versatile molecule.

## 2. Literature Search Methodology

We performed a literature search using databases such as PubMed, Web of Science, Scopus, ScienceDirect, and Google Scholar, with key terms including “melatonin”, “legumes”, “grain legumes”, “abiotic stress”, and “biostimulants”. The key terms were used alone or in combination to filter studies specifically related to grain legume species. To construct the tables, additional key terms were included for selected abiotic stress factors, such as “drought”, “salinity”, “metal”, “metalloids”, and “heat”. The methodology described in all selected articles was analyzed, and information regarding each stress treatment, type of melatonin application, concentration of melatonin solution, and the main physiological, biochemical, and molecular effects was extracted. We included only studies published in English. No restriction on publication date was applied.

## 3. Abiotic Stress Alleviation in Grain Legumes Through MT Applications

### 3.1. Drought Stress

Drought is one of the major abiotic stresses affecting plant metabolism, with significant impacts on the growth, development, and productivity of different species [30]. In addition to its ecophysiological consequences on forests and native species, from an agricultural perspective, its importance lies in the reduction of crop yields, thereby threatening global agrifood security [31]. Thus, it is crucial to establish strategies to maintain or increase yields in drought-affected regions to meet future food demands, considering a projected world population of 10 billion by 2050 [2]. Drought-affected areas are distributed across all five continents, and no improvement in this situation is expected in the coming years due to fluctuations in precipitation patterns exacerbated by climate change. Indeed, projected climate change models show increasingly severe drought scenarios regardless of whether greenhouse gas emissions increase or decrease, across much of the world [32]. Experiments performed in grain legume crops have shown a positive relationship between drought intensity and yield reduction; however, interspecific and varietal differences exist [33]. This broad genotypic and phenotypic variability has enabled the selection of more drought-tolerant varieties, which display differentiated metabolic responses under drought conditions. Some studies have reported that grain legume species respond to drought using at least two resistance mechanisms: avoidance and tolerance. Avoidance mechanisms are mediated by the efficient regulation of stomatal opening [34], whereas tolerance mechanisms involve osmotic adjustment, which maintains turgor and root growth, enabling water uptake under low soil water potential [35]. These mechanisms have been observed in both classical grain legume crops and woody legume species [36]. However, while stomatal regulation decreases CO_2_ fixation, negatively affecting photosynthesis and yield, osmotic adjustment mechanisms may be more energetically favorable [35]. In addition, drought induces profound changes in the primary and secondary metabolism of grain legumes [37,38]. Activation of metabolic pathways leading to the synthesis of osmoprotectant molecules can alter the allocation of photo-assimilates and other compounds to the developing grain, ultimately affecting its nutritional quality [39]. Another metabolic consequence of drought is the overproduction of reactive oxygen species (ROS), which generates redox imbalance at the cellular level, leading to thylakoid membrane damage through the oxidation of lipids and proteins, and impairing photosynthesis and other physiological functions [40].

One strategy to manage drought stress is the use of biostimulant formulations that enhance plant metabolic stress responses, such as the activation of enzymatic and non-enzymatic antioxidant machinery, and the induction of osmotic adjustment mechanisms [41]. Commonly used biostimulants include amino acids, peptides, seaweed polysaccharides, and silicon, among others [12,42]. Over the last decade, increasing evidence has supported the use of MT as a biostimulant to improve plant physiological functions under abiotic stress [21,43]. In grain legume species, MT has been applied to mitigate the detrimental effects of drought stress on plant physiology in soybean [28,44,45,46,47,48,49,50], mung bean [51], lentil [52], chickpea [53], and common bean [54,55]. Table 1 summarizes the main studies reporting the ameliorative effects of MT on drought-stressed grain legumes. A common response observed after MT application under drought conditions is an increase in enzymatic antioxidant activity, which decreases oxidative stress triggered by drought and improves the functionality of the photosynthetic machinery, ultimately enhancing biomass and yield (Table 1). In soybean, MT application has also been shown to affect the expression of genes involved in nitrogen metabolism, thereby influencing nitrogen accumulation and photosynthetic capacity [48]. In addition, the synthesis of osmoprotectant molecules, such as amino acids and soluble sugars, has been reported in several grain legume crops following MT treatment under drought stress.

### 3.2. Salinity Stress

Salinity stress is a major abiotic factor limiting crop yield, particularly in arid and semi-arid regions where salt concentrations are naturally high. Soil salinization can result from several factors, including the conversion of coastal habitats into agricultural lands, flooding, sea-level rise, and geological conditions, among others [56,57,58]. Salinity stress disrupts various physiological processes in grain legume crops, such as germination, growth, development, and nitrogen fixation [59]. In plants, two main stages of salt stress have been identified: the osmotic phase and the ionic phase. The osmotic and ionic stress imposed by salinity reduces cell expansion and, consequently, tissue growth, thereby affecting the yield and quality of grain legumes [60]. The first, early phase is associated with the effects of high salt concentrations outside the root zone, while the second refers to the toxic effects of salt accumulation within plant tissues [61]. Therefore, the molecular mechanisms of salt stress tolerance in plants are temporally and spatially separated. Elevated Na^+^ and Cl^−^ concentrations interfere with the assimilation of other nutrients such as N, P, B, K, Ca, Mg, Zn, Cu, and Fe, among others, inducing a nutritional ionic imbalance that impairs photosynthesis and other biochemical processes [59,62]. At the cellular level, salinity stress leads to an accumulation of toxic ions in the cytoplasm, disturbing ionic equilibrium and increasing ROS concentrations, which in turn causes the disintegration of cellular and organelle membranes. Such damage compromises photosystem functionality and reduces chlorophyll and carotenoid content, thereby impairing photosynthesis and productivity [63]. Most grain legumes are sensitive to salinity stress, which disrupts essential physiological functions throughout their phenology. Common responses to salinity stress in grain legumes include Na^+^ sequestration and exclusion from the cytoplasm, accumulation of osmoprotectant molecules (Pro, GB, GABA, PAs), enhanced antioxidant enzyme activity (CAT, POD, SOD, APX, GR, DHAR, and MDHAR), and hormonal regulation, all of which contribute to restoring ionic and oxidative homeostasis [60,64]. Various strategies have been used to alleviate salt stress in legume species, including chemical priming, inoculation with nitrogen-fixing bacteria, plant growth-promoting rhizobacteria, arbuscular mycorrhizal fungi, genetic manipulation, and the identification of salinity-adapted cultivars [65]. Most of these approaches focus on improving nutrient uptake, enhancing antioxidant machinery, and stimulating the osmoprotectant synthesis. In chemical priming, one of the most commonly used molecules is SA, which enhances photosynthesis and antioxidant responses, thereby reducing the effects of salinity in *Pisum sativum* [66] and *Vicia faba* [67]. By contrast, the use of MT to alleviate salinity stress has recently emerged as an alternative in several crops, including grain legumes. For instance, in soybean, exogenous MT applications induce the expression of genes involved in cell division, carbohydrate metabolism, fatty acid biosynthesis, and ascorbate metabolism [44], thereby counteracting the inhibitory effects of salt stress on gene expression. Similarly, in common beans, MT application to salt-stressed sprouts alleviated growth inhibition, increased antioxidant enzyme activity, and upregulated the expression of genes involved in the phenylpropanoid pathway [68]. Table 2 summarizes recent reports on MT use in grain legumes, highlighting their biochemical, molecular, and physiological effects under salinity stress.

### 3.3. Metal and Metalloid Stress

Due to their sessile nature, plants are often exposed to high concentrations of metals and metalloids in the soil, leading to a range of anatomical, physiological, and molecular responses. This exposure results from the widespread accumulation of these elements in soils and is largely associated with anthropogenic activities such as industrialization, excessive fertilizer use, and mining [79]. In addition to their toxic effects on crop physiology and implications for yield, the accumulation of metals and metalloids in plant tissues poses a threat to food security and human health due to their entry into the food chain [80,81]. While some of these elements are essential for plant metabolism, acting as cofactors in various enzymatic reactions, they become hazardous at high concentrations, inducing oxidative stress and cellular damage. Significant impacts on physiological processes such as transpiration, stomatal conductance, photosynthesis, and chlorophyll content, along with growth inhibition, have been associated with metal exposure in plants [82]. The toxicity of metals and metalloids has been reported in several species, with cadmium (Cd) being one of the most widely studied. The primary effects of Cd in plants include growth inhibition, chlorosis, impaired stomatal opening, disturbed water balance, reduced nutrient uptake, and decreased photosynthetic rates [83]. At the cellular level, Cd induces oxidative stress and ROS production, impairing enzymatic antioxidant machinery, with effects that vary depending on Cd concentration and plant species [84]. In legumes specifically, Cd exposure negatively affects germination rate, stem and primary root length, leaf number, lateral root development, and seedling dry weight in common bean [85], while reductions in roots and shoots’ dry weight have also been observed in soybean and lupine [86]. Likewise, reduced root biomass has been reported in peanut plants exposed to high concentrations of Fe [87]. Arsenic (As) toxicity has been described in soybean, where exposure leads to reduced seedling growth and root tip necrosis [88], while in *Cajanus cajan*, significant alterations in root growth and anatomy have been observed under As stress [89]. Some plant species exhibit mechanisms to cope with metals and metalloid stress by producing chelating molecules such as metallothioneins, phytochelatins, amino acids, nicotinamides, glutathione, and defensins [90,91].

The use of biostimulants to mitigate the detrimental effects of metal and metalloids on plant metabolism has been previously reviewed [92]. Among the most commonly used are microbial consortia, plant extracts, seaweed extracts, humic substances, and protein hydrolysates. These compounds enhance antioxidant responses in plants under metal/metalloid stress, thereby helping to restore oxidative homeostasis. In the case of MT, exogenous applications in tomato have been shown to reduce Cd root-to-shoot translocation and enhance sulfur (S) metabolism [93]. In wheat, MT alleviates Cd stress by increasing nitric oxide (NO) levels and decreasing oxidative stress [94]. In addition, reports describing the effects of MT under metal and metalloid stress in peanut, soybean, faba bean, and mung bean are summarized in Table 3, highlighting the main biochemical, molecular, and physiological responses. Overall, MT applications in grain legumes exposed to metal/metalloid stress have shown metal/metalloid-dependent responses, with multiple effects in ameliorating oxidative stress.

### 3.4. Heat Stress

It is well-known that heat stress events have increased due to climate change, leading to reduced plant growth and crop yields [100]. According to some studies, the average temperature on Earth’s surface increased by 1 °C between 1951 and 2012, with projections indicating a further increase of 1.5 °C between 2030 and 2052, between 2.5 and 4.8 °C by 2100 [101]. Therefore, heat stress is expected to become a critical factor for plants in the coming years. Heat stress negatively affects root and shoot growth, seed filling, photosynthesis, and protein synthesis, and induces the production of reactive oxygen species (ROS), which damage molecules and cellular organelles in plants [102,103,104,105]. In grain legume crops, negative impacts have also been reported under heat stress. Flowering has been identified as a particularly sensitive stage, with heat causing abnormal pollen grains and reducing pollen viability, pollen tube growth, and pollen germination [106,107]. Additionally, heat stress has a detrimental impact on pollen traits, thereby reducing yield components. For example, Wang et al. [102] reported that *Cicer arietinum* plants exposed to heat stress early in the flowering stage showed reductions in seed number per plant and seed size. Similarly, Sita et al. [108] found that heat stress reduced pollen tube elongation in *Lens culinaris*, leading to decreased grain yield. Conversely, Barros et al. [109] observed that heat stress induced flower abortion, reduced pollen viability and seed number, and decreased yield in *Vigna unguiculata*.

It has also been reported that grain legume crops respond to heat stress through tolerance and avoidance mechanisms, such as increased transpiration rates, development of waxy protective layers, synthesis of osmoprotectant molecules, activation of enzymatic and non-enzymatic antioxidants, and regulation of plant hormone levels [110,111,112]. In this context, positive responses have been observed following the exogenous application of certain biostimulants in grain legume crops exposed to heat stress. For instance, the exogenous application of proline, glycine betaine, salicylic acid, and abscisic acid enhanced plant growth and yield in *Cicer arietinum* [113,114,115]. Similarly, exogenous application of MT has been suggested to improve physiological processes in grain legume crops, thereby increasing growth and yield [116]. Table 4 summarizes studies on MT applications in grain legumes under heat stress, highlighting their main biochemical and physiological effects.

## 4. Integrating Molecular and Biochemical Insights into MT Functioning in Grain Legumes

The stress-mitigating effects of MT treatments have been studied in several grain legume species (Table 1, Table 2, Table 3 and Table 4), yielding results similar to those observed in other crops [121,122]. The favorable physiological effects of MT-treated plants appear to include both direct and indirect responses, involving enhanced antioxidant capacity triggered by MT. This enhancement is characterized by increased activity of antioxidant enzymes and higher levels of antioxidant compounds, such as flavonoids, phenolic compounds, proline, and melatonin itself (Table 1, Table 2, Table 3 and Table 4). MT is an intrinsic antioxidant molecule with a high capacity to scavenge ROS [123]. Owing to its amphipathic properties, it can cross biological membranes to broaden its targets [124]. Environmental stresses lead to an imbalance of reactive oxygen species (ROS), exposing plant membranes, organelles, and biomolecules to oxidative damage, thereby disrupting cellular homeostasis [125]. Superoxide (O_2_^−^) is one of the main ROS produced under abiotic stress, as a consequence of electron leakage in the electron transport chain (ETC) of both chloroplasts and mitochondria [126]. Additionally, hydrogen peroxide (H_2_O_2_) and hydroxyl radicals (OH) can form as a result of superoxide reactions [127]. High levels of ROS induce the expression of antioxidant enzymes (SOD, APX, CAT, POD, among others), along with the production of molecules derived from the phenylpropanoid pathway, such as phenolic compounds and flavonoids, to cope with cellular oxidative stress [128]. This activation of antioxidant machinery involves signal transduction and the expression of regulatory genes, which have been extensively studied in model plant species [129] but less explored in legumes. Thus, the fine-tuned regulatory role of MT in antioxidant machinery activation remains elusive. Two main questions therefore need to be addressed: (1) To what extent is the boost in antioxidant capacity a direct result of MT’s ROS scavenging? (2) Is MT directly involved in activating signaling pathways that regulate the transcription of antioxidant enzymes and molecules? The first question is difficult to answer due to experimental limitations, such as the inability to fully silence endogenous MT production in order to quantify ROS scavenging attributable solely to MT applications. For the second question, some insights have been reported in legumes. Interestingly, the effects of MT depend on its dose and mode of application, showing an active role in plant physiology at low concentrations across different organs [130]. Integrated transcriptomics and metabolomics studies have explored global responses and mechanisms triggered after MT applications under abiotic stress in common bean [68,72,73]. MT has been shown to increase the expression of genes related to cell wall metabolism in bean sprouts under salinity stress [73] and to enhance the expression of phenylpropanoid biosynthesis genes such as 4-coumarate-CoA ligase (*4CL*) and peroxidase (*POD*), along with lignin accumulation [68], suggesting a link between MT and cell wall reinforcement. In addition, under salt stress, MT treatment enhanced tryptophan metabolism and upregulated the expression of a putative transmembrane salt transport gene [72]. In soybean, available evidence under salt stress suggests that MT upregulates the expression of genes involved in cell division, photosynthesis, carbohydrate metabolism, fatty acid biosynthesis, and ascorbate metabolism, although no clear specific mechanism of action has been established [44]. In alfalfa (*Medicago sativa* L.), exogenous MT induces the transcription of genes involved in Ca^2+^ signaling, starch and sucrose metabolism, plant hormone signal transduction, and key transcription factors (C3Hs, MYBs, ERFs, and WRKYs), suggesting that MT plays an active role in starch and sugar metabolism and hormone-regulated signal transduction during salt stress [131]. Additionally, changes in the expression of calmodulin-like proteins (CLPs) in common bean roots after MT treatment confirm the interaction between the Ca^2+^ signaling pathways and MT in legumes [132]. The interaction between MT and calmodulin proteins has also been reported in other plant species [133,134], suggesting an indirect role of MT in regulating intracellular Ca^2+^ levels [135]. In *Vicia faba*, MT binds to calmodulin, altering its subcellular distribution. Furthermore, Ca^2+^ and melatonin treatments protect against As toxicity by enhancing antioxidant enzyme activity, regulating the AsA-GSH pathway, and activating plasma membrane H^+^-ATPase and Ca^2+^-dependent protein kinases [98].

**Figure 1:**
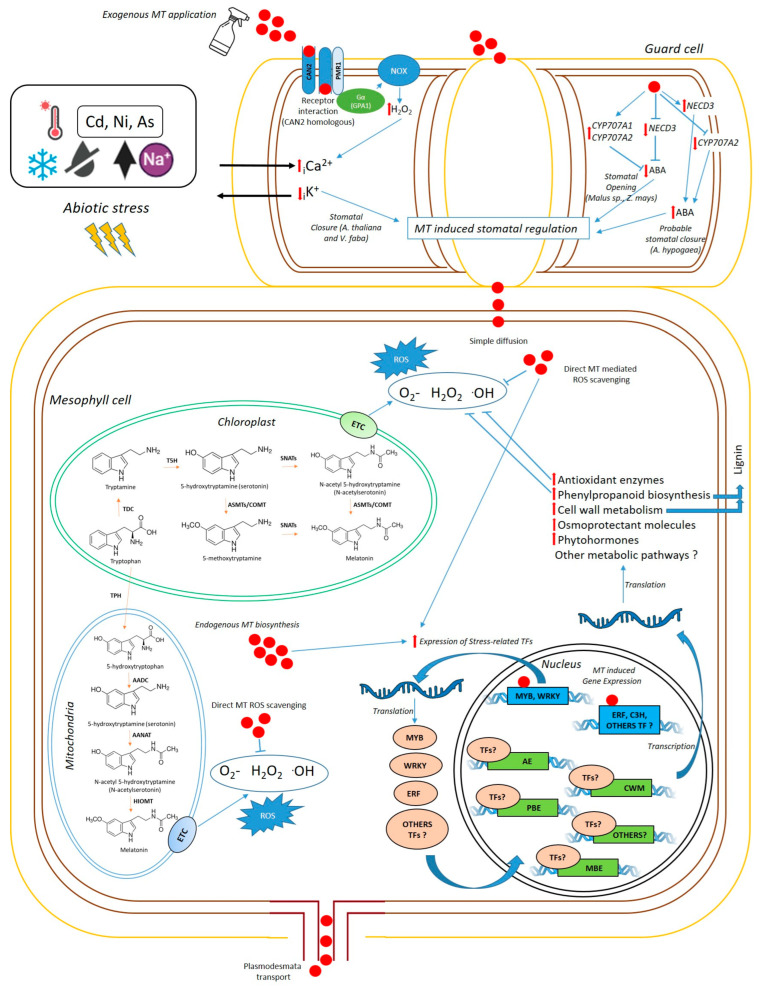
Simplified schematic representation of MT functioning in plant cells of grain legumes at the leaf level (Original figure). In *A. thaliana* and *V. faba,* MT induces stomatal closure in guard cells through interaction with the CAN2/PMR1 receptor. CAN2/PMR1 activates H_2_O_2_ production via NADPH oxidase (NOX) through Gα(GPA1), inducing Ca^2+^ influx and K^+^ efflux, thereby triggering stomatal closure. In other species (*Malus prunifolia*, *Malus hupehensis,* and *Zea mays*), MT induces downregulation of *NCED3* gene expression, decreasing ABA levels and promoting stomatal opening under drought. MT also upregulates ABA catabolism, inducing the expression of *CYP707A1* and *CYP707A2*. Otherwise, in *Araquis hypogaea*, MT induces an upregulation of *NCED3*, and a downregulation of *CYP707A2,* increasing ABA levels. MT can enter mesophyll cells by simple diffusion or be transported across plasmodesmata. MT can reduce oxidative stress either by directly scavenging reactive oxygen species (ROS) or by activating the expression of stress-related transcription factors (TFs) which enhance the expression of proteins involved in multiple metabolic pathways to ameliorate abiotic stress, such as antioxidant enzymes (AE), phenylpropanoid biosynthetic enzymes (PBE), and enzymes involved in cell wall metabolism (CWM). Additionally, exogenous MT induces the expression of melatonin biosynthetic enzymes (MBE), enhancing endogenous MT synthesis.

On the other hand, the endogenous biosynthesis and regulation of MT production remain under active investigation. Although MT biosynthetic pathways have been more thoroughly characterized in model species, they are less understood in legume crops [136]. The main substrate for MT biosynthesis is tryptophan, which is first converted to tryptamine by tryptophan decarboxylase (TDC) and subsequently to serotonin by tryptamine 5-hydroxylase (T5H). Finally, serotonin undergoes acetylation and methylation, catalyzed by serotonin N-acetyltransferase (SNAT) and N-acetylserotonin methyltransferase (ASMT), respectively, to form melatonin [129]. Alternatively, this last step can also be catalyzed by caffeic acid O-methyltransferase (COMT). Interestingly, the expression of genes encoding these enzymes (SNAT, ASMT, T5H, TDC) increased in pepper after MT treatment, under cold stress [137]. Similarly, the expression of *SNAT* and *ASMT* genes increased following MT treatment during temperature stress in soybean [119], suggesting that exogenous MT may induce endogenous MT biosynthesis under stress conditions. Additionally, MT biosynthetic enzymes have been implicated in autophagy, antioxidant signaling, and stress responses in cassava [138]. Although some enzymatic steps are shared among species, the specific genes, gene copy numbers, gene compartmentalization, or possible alternative enzymes involved in MT biosynthesis in legumes have not been fully identified. The MT biosynthetic pathway in plants can vary among species and environmental conditions, and it is localized in multiple cellular compartments, including the chloroplast, mitochondria, and cytoplasm [138,139,140]. This compartmentalization likely contributes to the pleiotropic effects of MT in plants. Figure 1 presents a simplified schematic representation of MT functioning, highlighting the most studied steps, main effects, and critical gaps that remain in grain legumes.

## 5. Biotechnological Prospects for MT Research in Grain Legumes

The available information on the genetic control of metabolic responses induced under abiotic stress conditions, together with access to molecular editing tools, opens opportunities to manipulate key metabolic pathways to enhance stress tolerance in crops [141]. However, in grain legume crops, the implementation of editing protocols is particularly challenging due to the recalcitrant nature of transformation efficiency and in vitro regeneration of legume species [142]. Nevertheless, advances using CRISPR/Cas9 editing have been reported mainly for soybean to alleviate abiotic stress such as drought [143,144,145] and salinity [146,147]. Additionally, editing or transformation/regeneration protocols have been implemented for chickpea [148], common bean [149], faba beans [150], and other legume species [151], expanding the possibilities for genome editing in legumes. Because of the pleiotropic nature of MT action, identifying specific genetic targets to enhance MT-mediated abiotic stress tolerance without affecting other pivotal cellular processes could be challenging. However, recent advances have opened the possibility of modifying target genes involved in endogenous melatonin biosynthesis or the regulatory control of tissue-specific melatonin production [152]. Likewise, modifying MT receptors in cellular organelles susceptible to oxidative damage could enhance MT efficiency or tissue-specific translocation [153]. Recently, in soybean, a 31-fold increase in melatonin content was achieved using a synthetic transcriptional regulator, showing the potential for melatonin bio-production and enhanced stress resilience in plants [154]. In this context, further efforts are needed to identify species-specific biosynthetic pathways, gene copy numbers and sequence variants, their cellular compartmentalization, as well as additional melatonin receptors in legume crop species.

Current breeding approaches, such as candidate-gene and genome-wide association studies (GWAS) and genomic selection, will play a major role in identifying and selecting genetic variants and, consequently, cultivars more responsive to melatonin applications, allowing us to explore the broad genetic diversity present in grain legume crops [132]. For instance, the analysis of 111 CLPs in common bean identified nine MT-responsive genes expressed in roots, providing valuable information about the tissue-specific expression of CLP in the context of MT signaling pathways [132]. In *Dianthus caryophyllus* L., an ornamental species, ten genes involved in MT biosynthetic pathways were identified by in-silico genome-wide analysis, with some of them showing a MT dose-dependent expression, opening opportunities for functional editing [155]. In maize, alleles associated with root traits were identified by exploring the natural variation of melatonin biosynthetic genes [156], thus providing key loci for breeding maize varieties with superior root systems. Additionally, a valuable trait for human health applications is the search for plant species with higher MT content. A recent study showed the potential of GWAS to identify genes associated with MT content in *Sesamum indicum* L., an oilseed species with high MT content, exposing its biotechnological potential [157]. Moreover, a negative regulator of melatonin content and signaling was identified using GWAS in a natural population of 228 cassava accessions (*Manihot esculenta*). *Melatonin accumulation 1* (*MA1*) encodes type 2C protein phosphatase 1 (PP2C1), which catalyzes the dephosphorylation of *MeRAV1/2* and *MeWRKY20,* two transcriptional regulators of the melatonin biosynthetic pathway. In addition, MePP2C1 dephosphorylates the phyto-melatonin receptor MePMTR1, repressing its binding to melatonin [158]. These studies highlight the importance of modern breeding approaches in MT research.

**Figure 2:**
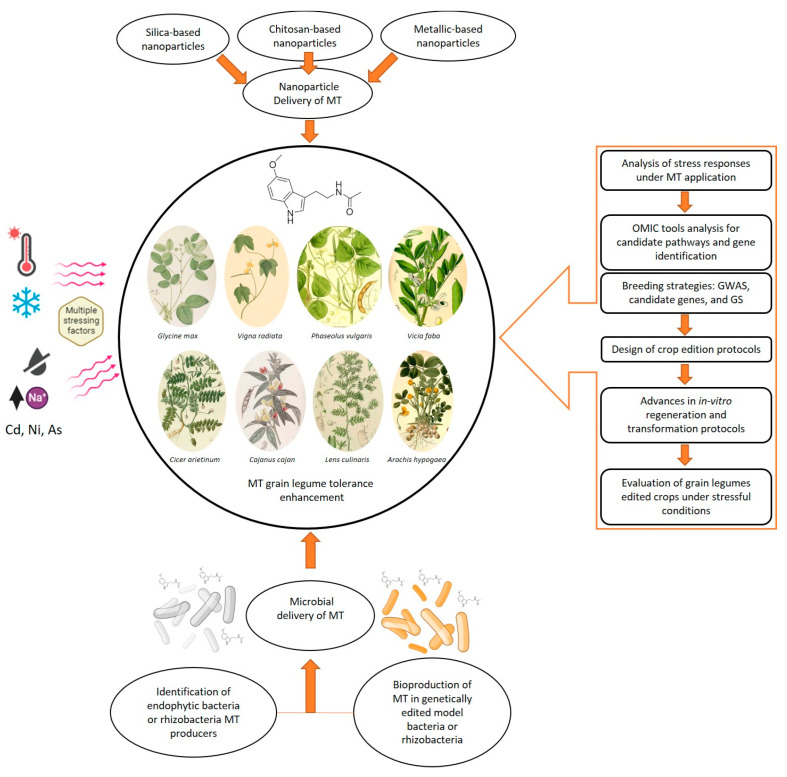
Schematic representation of a methodological workflow based on melatonin applications in grain legumes under stress, highlighting how modern breeding strategies, biotechnology tools, improved delivery protocols, and microbial MT production can support MT research for sustainable agriculture (Original figure). Grain legume illustrations were obtained from www.plantillustrations.org. GWAS: genome-wide association studies; GS: genome selection.

Complementary to the aforementioned approaches, another major technology gap in MT research is the development of efficient MT delivery systems. MT is light-sensitive [159] and can degrade if applied under high-light conditions. Therefore, developing nanoparticle-based delivery protocols to protect MT during field application is key to ensuring its functionality under abiotic stress conditions. Some advances have been made in this area for biomedical uses of MT, such as selenium-based nanoparticles, chitosan-based nanoparticles, solid lipid nanoparticles, and nanostructured lipid carriers [160]. For crop applications, an efficient delivery system based on mesoporous silica nanoparticles with or without chitosan improved Cd tolerance in rice, reducing Cd concentrations in rice leaves by 43% [161]. Other candidate delivery systems for MT applications in crops, including chitosan and metallic nanoparticles, have been previously reviewed [162]. Despite these advances, challenges such as cost, stability, and delivery specificity still need to be addressed. Moreover, the use of microorganisms such as endophytic bacteria and rhizobacteria as a low-cost and sustainable MT source for production and delivery has also been explored with promising applications [163,164]. Combined with advances in microbial editing tools and synthetic biology, this approach could revolutionize the biomanufacturing of MT, lowering production costs and improving shelf life and stability (Figure 2).

## 6. Conclusions

In this review, we have highlighted recent advances in MT research for grain legume crops, detailing key biochemical and molecular insights into the effects of MT under major abiotic stress conditions. Common responses were observed in grain legumes, regardless of species or stress type, including the enhancement of enzymatic antioxidant machinery and nitrogen metabolism, together with the induction of antioxidant and osmoprotectant molecules. These responses boost photosynthetic capacity, growth, and ultimately yield in several grain legume species. Despite numerous reports assessing the effects of MT on grain legumes under abiotic stress, significant scientific gaps remain in understanding MT mechanisms and the regulation of endogenous MT, which must be addressed in the future. Additionally, the application of OMICs, genome editing tools, and breeding strategies will facilitate the selection of cultivars more responsive to MT and the development of edited varieties to enhance grain legume tolerance to abiotic stress. Finally, ongoing efforts to improve MT delivery and stability—through nanoparticles and microbial production—are expected to ensure the sustainability of MT applications.

## Figures and Tables

**Table 1:** Studies on MT applications in grain legumes for alleviating drought stress.

Species	Stress Treatment	MT Application/ Concentration	Biochemical and Molecular Effects	Physiological Effects	Ref.
*Glycine max* L.	Watering was withheld for 12 days until 20% field capacity, and plants were maintained for 10 days.	Seed coating/50 and 100 µM.	Not assessed	No significant reduction in biomass under drought.	[44]
*Glycine max* L.	Added 15% *w*/*v* of PEG6000 to the irrigation solution to reach a water potential of −0.3 MPa.	Foliar spray and root inoculation/100 µM.	Increased activity of antioxidant enzymes SOD, POD, and CAT; reduced content of MDA.	Improved PSII efficiency, leaf area index, and yield.	[45]
*Glycine max* L.	Irrigation was reduced to 50% of field capacity, and plants were maintained under stress for 10, 17, and 24 days.	Foliar spray/100 µM.	Improvement of the antioxidant response, photosynthetic capacity, and content of amino acids	Promoted an overall increase in nitrogen accumulation and photosynthetic capacity.	[46]
*Glycine max* L.	Plants were subjected to water withholding until the soil moisture content reached 30–35% field capacity for 7 days.	Foliar spray or root irrigated for 5 days (5 mL of 50 or 100 µM MT, twice a day), before exposure to drought stress.	Increased activity of antioxidant enzymes and reduction of H_2_O_2_, MDA, and electrolyte leakage. Increase in JA, SA, and decrease in ABA, soluble sugar, and proline content.	Improved shoot length, root length, fresh weight, and dry weight.	[47]
*Glycine max* L.	Watering was withheld for 10 days until 50% of field capacity was reached, and plants were maintained under stress for 28 days. Samples were collected on days 18, 23, and 28.	Foliar spray/100 µM.	Increased expression of genes involved in nitrogen metabolism and enzyme activity, such as GOGAT, NR, GS, and GDH.	Promoted overall nitrogen accumulation in plants and increased photosynthetic capacity.	[48]
*Glycine max* L.	Watering was withheld until the leaf water potential reached −1.0 MPa.	Seed coating at 10, 30, 60, and 90 µM, and foliar spray at 30 and 50 µM.	Increased activity of antioxidant enzymes.	Increased germination speed index, root protrusion, and dry mass. Increased CO_2_ assimilation and net photosynthetic rate. Increased number of seeds, pods, and total seed mass.	[49]
*Glycine max* L.	Seedlings in V3 stage of fodder soybean. Drought conditions were achieved when the relative water content of soil reached 30%.	Foliar spray/50, 100, and 150 μM.	Reduced contents of H_2_O_2_, O_2_^−^ and MDA. Increased antioxidant capacity, and the content of osmoprotectants. Regulation of expression levels of genes associated with photosynthesis and the antioxidant defense.	Enhanced height, biomass and altered root morphology of fodder soybean seedlings.	[50]
*Glycine max* L.	Seedling in V3 stage of fodder soybean. Seven days of natural drought stress, induced using a weighing method until 30% relative water content of soil.	Foliar spray/100 μM.	Increased antioxidant capacity. Reduction in the endogenous ABA levels. Decreased expression of *GmABI5* transcription factor.	Increased drought resistance through ABA-independent pathways. Improved photosynthetic system. Changes in stomatal morphology.	[28]
*Vigna* *radiata* L.	Watering was withheld for 10 days at the flowering stage.	Seed treatment, foliar spray, and a combination of both/100 µM.	Enhanced activity of antioxidant enzymes such as SOD, CAT, and APX. Increase in metabolites involved in osmotic and ion homeostasis.	Improved physiological and yield-related traits.	[51]
*Lens* *culinaris* Medik.	Plants were maintained at 80% and 60% field capacity for 30 days.	Foliar spray/3 mM.	Increased the activity of antioxidant enzymes, soluble sugars, and antioxidant molecules.	Increased plant growth and biomass, photosynthetic pigments, and gas exchange parameters.	[52]
*Cicer* *arietinum* L.	Watering was withheld before the onset of flowering under field conditions.	Foliar spray/0, 100, and 200 µM in combination with 0, 3, and 6 µM of 24-epibrassinolide.	Improved enzymatic and non-enzymatic antioxidant activities such as CAT, SOD, PPO, APX, GPX, flavonoids, and carotenoids. Promotes the accumulation of proline, total soluble protein, and sugars.	Increased yield and its components, higher pigment content, enhanced oil and protein yield.	[53]
*Phaseolus vulgaris* L.	Seedlings were grown in Hoagland solution supplemented with 20% PEG6000 for 24 h.	Foliar spray/200 µM.	Increased expression of sn-Glycerol-3-phosphate-1-O-acyltransferase (*GPAT*) genes.	Not assessed.	[54]
*Phaseolus vulgaris* L.	Seedlings were grown in Hoagland solution supplemented with 20% PEG6000 for 24 h.	Foliar spray/200 µM.	Decreased levels of H_2_O_2_ and MDA, and increased activity of antioxidant enzymes. Changes observed in DNA methylation.	Not assessed	[55]
*Arachis hypogaea* L.	Drought was induced in 20-day-old seedlings using 10% PEG6000 for 4 days. Two varieties were assessed, a drought-sensitive and a drought-tolerant variety.	Seeds were imbibed with solutions of MT (5 µM, 10 µM, 25 µM, 50 µM and 100 µM).	Drought-sensitive variety showed an increase of endogenous MT content, antioxidant response, reduced expression and activity of the chlorophyll degrading enzymes and increase in chlorophyll content. Higher expression and activity of lipoxygenase (LOX) as genes involved in the synthesis of JA, including its content. Upregulation of *NCED3* and downregulation of *CYP707A2*, increasing ABA levels. No additional effects on drought stress responses of tolerant variety were observed.	Increased photosynthetic attributes.	[27]

**Table 2:** Studies on MT applications in grain legumes for alleviating salinity stress.

Species	Stress Treatment	MT Application/Concentration	Biochemical and Molecular Effects	Physiological Effects	Ref.
*Glycine max* L.	Plant seedlings were grown in soil saturated with 1% (*w*/*v*) NaCl for one to three weeks.	Seed coating/50 and 100 µM.	Upregulated expression of genes involved in cell division, photosynthesis, carbohydrate metabolism, fatty acid biosynthesis, and ascorbate metabolism.	Improved soybean growth.	[44]
*Glycine max* L.	Seeds were transplanted into soil contaminated with Cd^+^ and NaCl (50 mM).	Seeds treated with a combination of 1 mM calcium and 20 µM melatonin.	Improved germination, mineral content (Ca, P, K), and antioxidant properties, including DPPH activity, polyphenols, flavonoids, and SOD activity. Reduced MDA levels and enhanced proline content.	Improved germination and nutritional quality of soybean sprouts.	[69]
*Phaseolus vulgaris* L.	Ten days after sowing, salinity treatments of 4, 8, 10, and 16 dS m^−1^ NaCl were imposed in nutrient solution.	Seed coating/100 µM.	Increased activity of antioxidant enzymes such as SOD, POD, and APX. Facilitation of soluble protein synthesis. Improvement of K^+^/Na^+^ ratio.	Increased the shoot dry weight and seed yield.	[70]
*Phaseolus vulgaris* L.	Seedlings were exposed to 200 mM NaCl.	Incorporated by irrigation in Hoagland nutrient solution/150 µM.	Increased expression of genes *CuZnSOD*, *CAT1*, *APX*, *GR*, *PrxQ*, and *2-Cys-Prx*.	Increased photosynthetic capacity and enzymatic antioxidant response.	[71]
*Phaseolus vulgaris* L.	Seedlings grown in Hoagland nutrient solution plus 150 mM NaCl.	Incorporated by irrigation in Hoagland nutrient solution/100 µM.	Regulation of gene *Phvul 009G210332* and metabolites related to tryptophan decomposition.	Not assessed	[72]
*Phaseolus vulgaris* L.	Seeds germinated in a paper filter moistened with a 70 mM NaCl solution.	Seeds germinated in a paper filter moistened/100 µM.	Increased activities of antioxidant enzymes (SOD, POD, CAT, APX). Enrichment of the phenylpropanoid biosynthesis pathway, with 4-coumarate-CoA ligase (4CL) and peroxidase (POD) as critical enzymes.	Improved sprout length, surface area, volume, and average diameter.	[68]
*Phaseolus vulgaris* L.	Seeds germinated in a paper filter moistened with a 70 mM NaCl solution.	Seeds germinated in a paper filter moistened/100 µM.	Upregulation of cell wall pathway genes by at least 46%.	Increased the length, surface area, volume, and diameter of common bean sprouts.	[73]
*Phaseolus vulgaris* L.	Seedlings grown in Hoagland solution supplemented with 150 mM NaCl.	Foliar spray/200 µM.	Increased expression of sn-Glycerol-3-phosphate-1-O-acyltransferase (GPAT) genes.	Not assessed.	[54]
*Phaseolus vulgaris* L.	Seedlings were grown for 7 days in Hoagland solution supplemented with 150 mM NaCl.	Foliar spray/200 µM.	Decreased levels of H_2_O_2_ and MDA, and increased activity of antioxidant enzymes. Changes observed in DNA methylation.	Not assessed	[55]
*Phaseolus vulgaris* L.	Age of seedling not indicated. Fifteen days of salinity application (150 mM NaCl)	Type of application not indicated/25, 50, 100 µM.	Increased accumulation of polyphenol, proline, and ascorbic acid. Increased activity of SOD, POX, CAT, and APX. Reduced Na^+^ influxes, and increased K^+^ levels and K^+^/Na^+^ ratio.	Enhanced leaf canopy, total seedling length, and total seedling weight.	[74]
*Cicer arietinum* L.	Irrigation with 75 mM (to reach 4.68 dS m^−1^) and 100 mM (to reach 7.92 dS m^−1^) of NaCl	Foliar spray/50 and 100 µM.	Increased chlorophylls and carotenoid content. Decrease in oxidative markers (H_2_O_2_ and MDA) and increased CAT, SOD, and POD enzyme activities. Increased K^+^/Na^+^ and Ca^2+^/Na^+^ ratios in leaves.	Increased plant fresh weight, plant dry weight, root fresh weight, root dry weight, plant height, stem diameter, and leaf RWC.	[75]
*Cajanus cajan*	Watering with 200 mM NaCl.	Incorporated by irrigation in buffer solution/50 µM.	Promoted accumulation of luteoline by increasing expression of *F3’H-5* through the transcription factor *PCL1*.	Decreased MDA content and electrolyte permeability, retained chlorophyll content.	[76]
*Vicia faba* L.	Watering with 75 and 150 mM NaCl	Foliar application of 50 and 100 µM at 35 to 40 days after sowing.	Decrease the Na accumulation in plant tissues. Increased expression of genes encoding antioxidant enzymes (CAT, GR, Fe SOD, Cu-Zn SOD) and their antioxidant enzymatic activity. Higher content of AsA and GSH. Increased synthesis of glycine betaine, phenol, and proline.	Production of photosynthetic pigments was retained. Improved photosynthetic parameters.	[77]
*Vicia faba* L.	Fifteen days after sowing, seedlings were watered with diluted seawater at low and high concentrations (3.85 dS/m and 7.69 dS/m, respectively)	Seed soaked with 100 and 500 µM of MT.	Improved photosynthetic pigments, total carbohydrate, total phenolic content, indole acetic acid, K^+^, Ca^+2^, and reduced levels of compatible solutes, Na^+^ and Cl^−^ contents in leaf tissues of plants irrigated with diluted seawater.	Improved growth parameters and RWC. Treatment with 500 µM had a more pronounced effect.	[78]

**Table 3:** Studies on MT applications in grain legumes for alleviating metal and metalloid stress.

Species	Stress Treatment	MT Application/Concentration	Biochemical and Molecular Effects	Physiological Effects	Ref.
*Arachis* *hypogaea* L.	Three-week-old peanut seedlings watered with 1/2 Hoagland solution containing 100 μM CdCl_2_ for 21 days.	Watering with 1/2 Hoagland solution containing 50 μM.	Enhanced metabolism of linolenic acid, glutathione (GSH), and phenylpropanoid (lignin). Upregulation of *AhNHL* gene, promoting phenylpropanoid biosynthesis and GSH metabolism.	Enhanced development of the Casparian strip in the root cell wall. Decreased Cd accumulation in roots, shoots, and seeds by 40–60%, promoting antioxidant capacity.	[95]
*Glycine max* L.	NiO nanoparticles were applied to the soil at 100 mg kg^−1^	Foliar spray/50, 75, and 100 μM.	Enhanced levels of phytohormones (ABA, JA, SA, and GA4) and secondary metabolite production. Enhanced SOD, POD, CAT, and APX activities. Upregulated POD, CAT, and APX gene expression. Enhanced antioxidant and oxidative enzymes. Improved N_2_-assimilation enzymes (UE, NR, GS, GOGAT, GDH).	Protection of the photosynthetic pigments and Ni uptake reduction. Decreased Ni content in root and shoot. Improved soybean seedlings’ resilience by restoring growth, balancing ion accumulation, and reducing ROS production. Improved nodule formation and N_2_ content.	[96]
*Glycine max* L.	At 21 DAS, soybean seedlings were exposed to 50 μM of arsenic (NaAsO_2_) through the soil.	Around 30 DAS, the foliar portion of soybean seedlings was treated with MT and ZnO-nanoparticles in the morning and evening, respectively, for 5 days (30–35 DAS)/100μM.	Decreased oxidative damage by enhancing the activity of the enzymatic antioxidant system and proline content. Detoxification of the H_2_O_2_, MDA, and ROS levels in As-stressed plants	Increased photosynthesis efficiency and growth of the soybean plants.	[97]
*Vicia faba* L.	Plants were irrigated with a nutrient solution supplemented with 5 µM of Na_3_AsO_4_. The treatment was applied to 7-day-old plants every three days up to 34 days.	Irrigated with a nutrient solution supplemented with 50 µM. MT was applied to 7-day-old plants every three days up to 34 days.	Increased expression of ATP synthase, Ca^2+^-ATPase, Ca^2+^-DPKase exchangers, Hsp17.6, and Hsp40. Increased total soluble carbohydrates, cysteine, and proline accumulation with increased *P5CS* and decreased Pro-degrading enzyme.	Applied synergistically with Ca^2+^ suppressed cellular programmed death features. Enhanced gas exchange parameters and photosynthetic enzymes.	[98]
*Vigna* *radiata* L.	Seedlings (two-leaf stage) were treated with 200 μM CdCl_2_ for 48 h.	Seedlings (two-leaf stage) treated with 30 μM MT for 48 h.	Improved activity of anaplerotic enzymes (involved in the TCA cycle). Enhanced activity of nitrate reductase, nitrite reductase, and glutamine synthetase.	Enhanced Cd tolerance. Higher levels of ammonium and their subsequent assimilation into amino acids and proteins.	[99]

**Table 4:** Studies on MT applications in grain legumes for alleviating heat stress.

Species	Stress Treatment	MT Application/Concentration	Biochemical and Molecular Effects	Physiological Effects	Ref.
*Phaseolus vulgaris* L.	Study conducted in field conditions under high temperatures during the reproductive stage (over 40 °C)	Foliar spray at 15, 30, and 45 days after seed sowing/50 and 100 μM. This was combined with Mg applications in soil.	Protein content was increased from 22.54% in the control to 23.98%. Mg content in seeds was up to 0.84% under this treatment against 0.52% in the control.	Melatonin 100 µM under Mg (28.57 kg ha^−1^) increased plant height to 65.46 cm, chlorophyll content to 43.41 SPAD units, and seed yield per plant to 26.4 g from 18.1 g in the control.	[117]
*Cajanus Cajan*	One-month-old plants were grown in pots with soil. After MT pretreatment, T° was raised from 23 °C to 42 °C.	Plants were watered with 50 μM MT in buffer solution (0.1% ethanol in water) every other day for four times.	Increased expression of *CcF3’H-5,* a gene involved in luteolin biosynthesis.	Decreased MDA content and electrolyte permeability, and restored chlorophyll content.	[76]
*Glycine max* L.	At the V2 stage (when second trifoliate leaves start developing), plants were exposed to heat stress from 24 °C to 42 °C for 3 and 7 days.	Five days after transplanting, plants were pretreated with 30 mL of 100 μM MT, twice daily, for 6 days in the root zone.	Increased phenolic, flavonoids, proline, endogenous MT, and polyamines. Reduced ABA content, downregulation of *gmNCED3*, and upregulation of catabolic genes (*CYP707A1* and *CYP707A2*). Increased SA and upregulated expression of the *PAL2* gene. Induced expression of gmHsp90 and the heat shock transcription factor (*gmHsfA2*).	Increased plant growth, photosynthetic pigments (Chl a and Chl b), and reduced oxidative stress.	[118]
*Glycine max* L.	Soybean cell cultures were grown at 16, 24, and 32 °C.	After day 12 of culture, different combinations of melatonin/serotonin solutions were applied, with a maximum of 100 μM each.	Serotonin and melatonin improved isoflavone content. An overexpression of isoflavones, melatonin, cell division, ethylene biosynthesis genes, and transcription factors was observed.	Serotonin and melatonin increased biomass production during temperature stress.	[119]
*Vigna* *radiata* L.	Seedlings (five days old) were exposed to two heat stress treatments: (1) 40 °C and (2) 42 °C for 3 h.	Seeds were treated with different MT concentrations from 20 µM to 100 µM for 6 h.	80 and 100 µM MT doses showed higher superoxide dismutase and catalase activities with reduced oxidative stress.	High survival percentage of plants; high shoot and root growth.	[120]
*Vigna* *radiata* L.	The stress was imposed for 10 days at the flowering stage. T° was increased from 32 °C to 34 °C and 36 °C in an open-top chamber using an infrared heater.	Seed treatment, foliar spray, and a combination of both/100 µM.	Enhanced activity of antioxidant enzymes such as SOD, CAT, and APX. Increased metabolites involved in osmotic and ion homeostasis.	Improved physiological and yield-related traits.	[51]

## Data Availability

No new data were created or analyzed in this study. Data sharing is not applicable to this article.

## Abbreviations

The following abbreviations are used in this manuscript:

MT: melatonin, CRISPR: clustered regularly interspaced short palindromic repeats, ABA: abscisic acid, SA: salicylic acid, JA: jasmonates, GA: gibberellins, CK: cytokinins, BR: brassinosteroids, SL: strigolactones, ROS: reactive oxygen species, SOD: superoxide dismutase, POD: peroxidase, CAT: catalase, APX: ascorbate peroxidase, GPX: glutathione peroxidase, PPO: polyphenol oxidase, MDHAR: monodehydroascorbate reductase, DHAR: dehydroascorbate reductase, GR: glutathione reductase, PEG: polyethylene glycol, MDA: malondialdehyde, Pro: proline, GB: glycine betaine, GABA: γ-aminobutyric acid, PAs: polyamines, GOGAT: glutamine oxoglutarate aminotransferase, NR: nitrate reductase, GS: glutamine synthetase, GDH: glutamate dehydrogenase, DPPH: 2,2-diphenyl-1-picrylhydrazil, AsA: ascorbate, GSH: glutathione, GA4: gibberellin, UE: urease, DAS: days after sowing, ETC: electron transport chain, CLPs: calmodulin-like proteins, TDC: tryptophan decarboxylase, T5H: tryptamine 5-hydroxylase, SNAT: serotonin N-acetyltransferase, ASMT: N-acetylserotonin methyltransferase, COMT: caffeic acid O-methyltransferase, GWAS: genome-wide association studies.
